# Assessment of the Quality and Readability of Online Information on Thumb-Sucking Habit in Children

**DOI:** 10.7759/cureus.91585

**Published:** 2025-09-04

**Authors:** Asim Almarhoumi, Abdullah M Alharbi, Khalid N Alturki, Ahmad K Alsaedi, Muath S Alassaf

**Affiliations:** 1 Preventive Dental Sciences, Taibah University, Madinah, SAU; 2 Dentistry, National Guard Health Affairs Hospital, Taif, SAU; 3 Dentistry, Taibah University, Madinah, SAU; 4 Oral and Maxillofacial Surgery, Saudi Ministry of Health, Madinah, SAU

**Keywords:** discern, hon seal, internet health education, jama, online health information, readability, thumb-sucking habit

## Abstract

Aim: This study aimed to evaluate the quality and readability of online information on thumb-sucking habits among children, using the DISCERN instrument, Health on the Net (HON) seal, and Journal of American Medical Association (JAMA) benchmarks.

Methods: A systematic search was conducted on Google, Yahoo, and Bing for “thumb-sucking habit”. A total of 450 websites were screened; irrelevant content, duplicates, and heavily commercial or video-only sites were excluded, resulting in 143 sites. The DISCERN tool, with 16 questions rated from 1 to 5, assessed information quality, while the HON seal verification checked for HON compliance. JAMA benchmarks evaluated authorship, attribution, disclosure, and currency, and readability was assessed using Flesch Reading Ease, Flesch-Kincaid Grade Level, and SMOG (Simple Measure of Gobbledygook).

Results: The DISCERN mean score value was 2.73 (±0.57) out of 5, indicating moderate quality. Most sites were non-profit (86.01%), followed by commercial (7.69%), and university/medical centres (6.29%). DISCERN highlighted strengths in relevance and balance but weaknesses in source citations and references. Only 12 sites displayed a HON seal. Readability varied: university/medical centres scored the highest, whereas commercial websites, despite showing relatively higher DISCERN scores, had lower readability, making the information less accessible to the general public.

Conclusion: This study revealed that online information on thumb sucking is of moderate quality, with notable differences across website types. Clinicians should guide patients toward non-profit and HON-certified sites for more reliable resources. Enhancing transparency, citation practices, and readability remains essential to support informed health decisions regarding thumb-sucking habits.

## Introduction

In the age of information, the Internet has become an important and rapid source of health-related advice and knowledge. While this has empowered patients and caregivers to make informed decisions, it has also raised concerns regarding the accuracy, quality, and reliability of online health information. The quality of online information, particularly in the domain of dental and paediatric health, can have a significant impact on health decisions. Thumb sucking is a common behaviour among infants and toddlers, and it is imperative to assess the quality and readability of the information available online on this topic. It is a natural reflex in infants and young children who derive comfort and security from placing their thumbs or other fingers in their mouths [1]. This behaviour is exhibited by approximately 50% of infants by the age of one and can commence as early as in the womb. It typically diminishes as the child grows older, typically ceasing by the ages of two to four. Generally, children cease this behaviour naturally at approximately 3.8 years of age [2]. Nevertheless, this behaviour persists in some children throughout their preschool years and beyond [1].

Several studies have investigated the prevalence of thumb sucking in Saudi Arabia. A cross-sectional study conducted using a survey questionnaire and clinical research has shown that thumb sucking is common among preschool children, with no significant differences based on sex, birth order, or family income [3,4].

Various factors influence the quality of online health information. Some health-related websites prioritize visibility and market reach, which may compromise the accuracy and transparency of their content [5]. Eysenbach et al. emphasized that an astonishing 70% of health-related websites exhibited significant issues with quality [6]. The main obstacle associated with online healthcare content lies not in its quantity but rather in its credibility and accuracy. To address these difficulties, various tools such as DISCERN, the Health on Net (HON) seal code, and the Journal of American Medical Association (JAMA) benchmarks have been developed to assess the credibility of online information.

A thorough review of the literature up to 2015 identified only one study evaluating the quality of online information on thumb-sucking habits, using only the DISCERN and HONcode tools. This highlights the limited research in this area and underscores the need for further investigation. The overall quality of content was moderate, with few sites meeting high standards [7]. Therefore, this study aimed to evaluate the quality and readability of up-to-date online information in the English language on thumb-sucking habits. We DISCERN, JAMA, and HONcode benchmarks to assess the quality of information [8-12]. These findings highlight gaps in online information and can guide efforts to improve its quality for key stakeholders.

## Materials and methods

Search strategy

To obtain a comprehensive understanding of online English information pertaining to thumb-sucking habits, we performed a systematic web search. This entailed clearing browser cookies and activating incognito mode to avoid biased search results. Using prominent search engines such as Google, Yahoo, and Bing, searches were conducted using the term “thumb-sucking habit” and variations of the key term. We reviewed the top 15 result pages from each search engine, yielding a total of 450 websites per language. All sites were evaluated within a strict 24-hour period to minimize the influence of dynamic search algorithms, which can change search results over time. This approach ensures that all websites included in the analysis reflect a consistent snapshot of the available online information, enhancing the reliability and reproducibility of the study.

Websites that were freely accessible, primarily in English, and provided an in-depth analysis of thumb sucking were included. Exclusion criteria encompassed websites that were not mainly in English, offered only superficial mentions of thumb sucking, or lacked substantial content. Additionally, audio-visual sources, derivative content, and predominantly commercial sites were excluded.

Quality assessment

To evaluate the quality of the information, DISCERN, HONcode, and JAMA tools were employed. For the HONcode evaluation, an official HONcode extension into the Google Chrome browser was integrated. This extension verifies a website's compliance with the HONcode criteria and provides a one-year certificate if it satisfies the requirements. All websites that met the HONcode standards were cross-checked on the HONcode website.

The DISCERN tool, developed by Charnock et al., is widely recognized for its ability to critically appraise the quality of written health information [12]. It consists of 16 questions divided into three sections: the first eight assess the publication’s reliability, questions 9 to 15 evaluate the specifics of the treatment, and the final question provides an overall quality rating. Each question is scored on a scale from 1 (low quality) to 5 (high quality), and the cumulative score allows publications to be categorized into low, moderate, or high quality, reflecting their trustworthiness and utility for readers.

The JAMA benchmarks, introduced by Silberg et al. in 1997, are a set of criteria designed to help users identify credible online health information. These benchmarks evaluate the quality of content by assessing four key areas: authorship (who is responsible for the content), attribution (proper citation of sources), currency (how up-to-date the information is), and disclosure (transparency of conflicts of interest or funding) [8].

The tools employed in this study, DISCERN, JAMA benchmarks, and the HONcode browser extension, are publicly accessible and free to use for academic research and educational purposes.

To ensure the scoring reliability of all tools used, two assessors systematically calibrated and conducted the evaluations, and their average scores were used to categorise the results. If any inconsistencies arose between assessors, a third assessor was involved in resolving them.

Readability assessment

A readability calculator tool was created using Python in VSCode to gauge the ease with which website content can be read. Tools such as Flesch Reading Ease Score (FRES), Flesch-Kincaid Grade Level (FKGL), and SMOG (Simple Measure of Gobbledygook) were employed [9,10]. The acceptable readability level was determined as FKGL and SMOG scores less than 7, and FRES greater than or equal to 80 [11]. The readability assessment tools (FRES, FKGL, and SMOG) are freely available [9,10,11].

Statistical analysis

Data were collected and recorded using Microsoft Excel (Microsoft Corporation, Redmond, USA). Descriptive statistics, including means, standard deviations, and frequencies, were calculated. Inferential analyses were performed using chi-square tests, t-test, and ANOVA test to examine associations between categorical variables, utilizing IBM SPSS Statistics, version 26.0 (IBM Corp., Armonk, USA).

Ethical considerations

For this study, only publicly accessible data that did not contain any personal identifiers, confidential information, or other sensitive materials pertaining to individual patients or study participants were utilised. Given the nature of our study design, it was not necessary to obtain informed consent or undergo formal ethical review procedures typically required for research involving human subjects.

## Results

**Figure 1:**
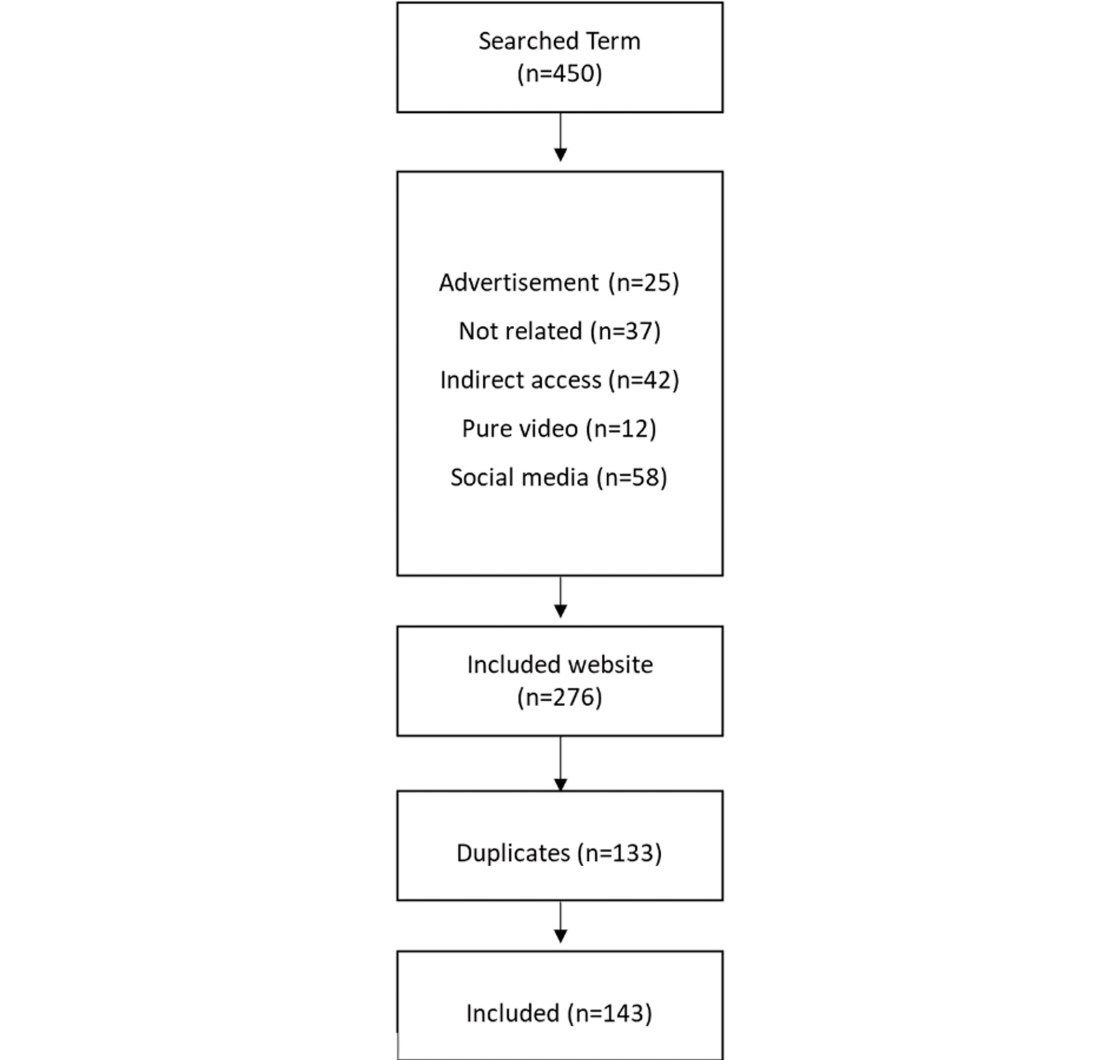
Flowchart of the search strategy

A total of 450 websites were initially identified. After excluding 175 for advertisements, irrelevant content, indirect access, video-only material, and social media, 276 remained. Of these, 133 were duplicates, yielding a final sample of 143 websites for analysis (Figure 1).

Table 1 summarises categorisation by affiliation, specialisation, content type, and presentation. The majority were affiliated with non-profit organisations (86.01%), followed by commercial entities (7.69%) and university/medical centres (6.29%). None were exclusively specialised in the topic of interest.

**Table 1:** Categorization of websites based on affiliation, specialization, content type, and content presentation (n = 143) Descriptive statistics are reported. No inferential tests were applied.

Category	Criteria	Number of websites	Percentage
Affiliation	Commercial	11	7.69%
	Non-profit organization	123	86.01%
	University/medical centre	9	6.29%
Specialization	Exclusively related	0	0.00%
	Partly related	143	100.00%
Content type	Medical facts	140	69.65%
	Clinical trials	2	0.99%
	Human interest stories	4	1.99%
	Question and answer	55	27.36%
Content presentation	Image	22	70.97%
	Video	9	29.03%
	Audio	0	0.00%

Medical facts constituted the predominant content type (69.65%), with question-and-answer formats accounting for 27.36%. Human interest stories (1.99%) and clinical trial information (0.99%) were infrequent. Regarding presentation format, images were most common (70.97%), followed by videos (29.03%); no site provided audio content.

DISCERN evaluation (Table 2) demonstrated variable quality across websites. The highest average scores were observed for relevance (Q3: 4.97, SD = 0.14) and provision of balanced, unbiased information (Q6: 4.72, SD = 0.52). The lowest scores were for explicit source citation (Q4: 1.72, SD = 0.91) and provision of additional sources (Q7: 1.79, SD = 0.64). The overall mean DISCERN rating was 2.73 (SD = 0.57), indicating moderate quality.

**Table 2:** Means and standard deviation scores for DISCERN instrument domains (n = 143) One-sample t-test (test value = 3) was used [12].

Domain	DISCERN question	Mean (SD)	Max.	Min.	Test statistics (t-test): t(df=142)	p-value
Reliability	Q1. Explicit aims	1.84 (1.58)	5.0	1.0	-8.49	<0.001
	Q2. Aims achieved	1.84 (1.59)	5.0	1.0	-8.43	<0.001
	Q3. Relevance	4.97 (0.14)	5.0	4.0	45.24	<0.001
	Q4. Explicit sources	1.72 (0.91)	5.0	1.0	-13.08	<0.001
	Q5. Explicit date	3.43 (1.09)	5.0	1.0	4.46	<0.001
	Q6. Balanced and unbiased	4.72 (0.52)	5.0	3.0	21.71	<0.001
	Q7. Additional sources	1.79 (0.64)	4.0	1.0	-20.61	<0.001
	Q8. Areas of uncertainty	2.01 (0.55)	3.5	1.0	-18.40	<0.001
Treatment options	Q9. How treatment works	2.73 (0.57)	4.0	1.5	-3.90	<0.001
	Q10. Benefits of treatment	2.53 (0.90)	4.5	1.0	-6.09	<0.001
	Q11. Risk of treatment	1.99 (0.66)	3.5	1.0	-17.11	<0.001
	Q12. Effects of no treatment	1.79 (0.67)	3.0	1.0	-20.10	<0.001
	Q13. Effects on quality of life	2.99 (1.05)	5.0	1.0	-0.10	0.92
	Q14. All alternatives described	1.92 (0.68)	3.5	1.0	-18.21	<0.001
	Q15. Shared decision	2.35 (0.87)	4.5	1.0	-7.07	<0.001
Overall rating		2.73 (0.57)	4.0	1.5	-6.28	<0.001

Further analysis by affiliation is presented in Table 3. Non-profit organisations achieved the greatest compliance with JAMA benchmarks, with 61 meeting two criteria and 10 meeting three. Only 12 websites attributed sources appropriately, and none disclosed conflicts of interest. Currency was maintained in 126 websites. Differences in JAMA compliance across affiliations were not statistically significant (p > 0.050).

**Table 3:** Quality and readability assessment based on JAMA benchmarks, i.e., authorship, attribution, disclosure, and currency, across website affiliations reported as frequency and percentage (n = 143) Test statistics were calculated using the chi-square test for independence (χ²(6) = 2.66, p = 0.85) [8].

Variable	Variable type	Commercial	University/medical centre	Non-profit organization	Total	Test statistic (χ²)	p-value
Number of achieved JAMA items per website	None	1 (9.09%)	1 (9.09%)	9 (81.81%)	11	χ²(6) = 2.66	0.85
	One	4 (7.69%)	5 (9.61%)	43 (82.69%)	52		
	Two	5 (7.35%)	2 (2.94%)	61 (89.70%)	68		
	Three	1 (8.33%)	1 (8.33%)	10 (83.33%)	12		
JAMA items	Authorship	8 (9.3%)	3 (3.48%)	75 (87.20%)	86	χ²(2) = 3.54	0.17
	Attribution	1 (8.33%)	1 (8.33%)	10 (83.33%)	12	χ²(2) = 0.18	0.91
	Disclosure	0 (0.0%)	0 (0.0%)	0 (0.0%)	0	N/A	N/A
	Currency	8 (6.34%)	8 (6.34%)	110 (87.30%)	126	χ²(2) = 2.67	0.26

Table 4 compares DISCERN scores and readability by affiliation. Commercial websites demonstrated the highest mean overall DISCERN score (45.0, SD = 9.6; 95% CI: 42.3-47.7), followed by non-profit organisations (40.5, SD = 6.5; 95% CI: 38.6-42.4) and university/medical centres (37.4, SD = 5.0; 95% CI: 36.0-38.8), though these differences were not statistically significant (p = 0.080). Reliability scores followed a similar trend, with commercial websites achieving the highest values (23.5, SD = 5.8; 95% CI: 21.9-25.1, p = 0.240). In contrast, treatment information scores differed significantly (p = 0.040), favouring commercial websites (18.4, SD = 4.5; 95% CI: 17.1-19.7) over non-profits (15.4, SD = 3.8; 95% CI: 14.3-16.5) and university/medical centres (14.3, SD = 3.7; 95% CI: 13.3-15.3).

**Table 4:** Comparison of DISCERN quality scores and readability across website affiliations using FRES, FKGL, and SMOG indices (n = 143) FRES: Flesch Reading Ease Score; FKGL: Flesch-Kincaid Grade Level; SMOG: Simple Measure of Gobbledygook p-values are from one-way ANOVA across website affiliations. *Statistically significant results were found for treatment (F(2,140) = 3.31, p = 0.04), FRES (F(2,140) = 3.13, p = 0.047), and FKGL (F(2,140) = 3.54, p = 0.032) [9,10,11].

Variable	Commercial, mean (±SD), 95% CI	University/medical centre, mean (±SD), 95% CI	Non-profit organization, mean (±SD), 95% CI	Test statistic (F)	p-value
Overall	45.0 (9.6), 42.3-47.7	37.4 (5.0), 36.0-38.8	40.5 (6.5), 38.6-42.4	F(2,140) = 2.57	0.08
Reliability	23.5 (5.8), 21.9-25.1	20.5 (2.9), 19.7-21.3	22.3 (4.1), 21.1-23.5	F(2,140) = 1.43	0.24
Treatment	18.4 (4.5), 17.1-19.7	14.3 (3.7), 13.3-15.3	15.4 (3.8), 14.3-16.5	F(2,140) = 3.31	0.04*
FRES	71.1 (36.8), 60.7-81.5	96.3 (9.6), 93.6-99.0	87.4 (19.3), 81.9-92.9	F(2,140) = 3.13	0.047*
FKGL	16.7 (16.7), 12.0-21.4	4.6 (4.7), 3.3-5.9	12.6 (41.9), 0.6-24.6	F(2,140) = 3.54	0.032*
SMOG	3.3 (0.5), 3.2-3.4	3.5 (1.2), 3.2-3.8	3.6 (0.9), 3.3-3.9	F(2,140) = 1.24	0.29
Words	874.4 (635.4), 694.6-1054.2	542.9 (288.0), 461.4-624.4	615.0 (339.1), 518.1-711.9	F(2,140) = 0.64	0.53
Sentences	30.8 (28.3), 22.8-38.8	41.2 (41.4), 29.5-52.9	23.8 (17.6), 18.8-28.8	F(2,140) = 1.09	0.34

**Figure 2:**
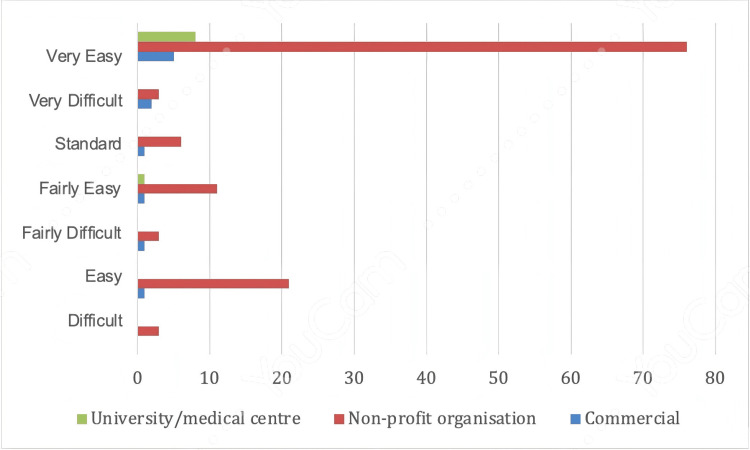
The Flesch Reading Ease Score (FRES) difficulty categories for the evaluated websites (n = 90) Image credits: Created using YouCam (CyberLink Corp., Taipei, Taiwan) by Abdullah Mohammed Alharbi.

Significant variation was observed in readability measures. The FRES differed across affiliations (p = 0.050), with commercial websites scoring the lowest (71.1, SD = 36.8; 95% CI: 60.7-81.5) and university/medical centres the highest (96.3, SD = 9.6; 95% CI: 93.6-99.0) (Figure 2). The FKGL also varied significantly (p = 0.030), with commercial websites presenting the greatest complexity (16.7, SD = 16.7; 95% CI: 12.0-21.4). SMOG scores, content length, and sentence count did not differ significantly across groups (p > 0.050).

Overall, non-profit organisations dominated in number and in achieving JAMA criteria. Commercial websites provided more comprehensive DISCERN and treatment information but demonstrated lower readability. University/medical centres offered the highest readability yet ranked lower in quality metrics, highlighting distinct strengths and limitations across affiliations.

## Discussion

Persistent thumb sucking in children can significantly impact craniofacial development, leading to dental malocclusions such as protrusion of teeth, open bite, and high, narrow palates. These changes may contribute to speech impediments and, in severe cases, facial deformities. Additionally, prolonged thumb-sucking habits increase the risk of oral infections and can have psychosocial consequences, such as teasing and reduced self-esteem. A range of interventions, from behavioural strategies to habit-breaking appliances, have been shown to reduce these habits [1,8,13,14].

Given the clinical and social implications, access to accurate and comprehensible online information is crucial for parents and caregivers. However, the reliability of such content varies considerably, and previous research has highlighted concerns regarding its overall quality [7]. The present study aimed to evaluate the quality and readability of English-language online resources on thumb-sucking habits [15].

Our findings align with the previous literature, confirming that the quality of online information remains moderate [5,12,16]. While most content was relevant and balanced, deficiencies in source citation and referencing were evident, reducing transparency and credibility. Similar limitations have been reported in other health-related topics, suggesting a broader trend in online health information quality.

Non-profit organisations represented the largest proportion of websites, which may suggest a degree of credibility; however, the limited representation from academic and medical centres indicates a gap in specialist-driven resources [15]. None of the websites were exclusively focused on thumb sucking, underscoring the lack of dedicated educational platforms for this common habit. Most content focused on medical facts and question-and-answer formats, with images as the primary visual aid. The absence of audio content highlights an opportunity to improve accessibility for diverse users [17,18].

In terms of quality assessment, commercial websites scored the highest on overall DISCERN and treatment-related information, suggesting a more structured approach to presenting management options. However, these sites demonstrated the lowest readability, potentially limiting their usefulness for the general public. In contrast, university and medical centre websites were the most readable but offered less comprehensive treatment information. Non-profit organisations showed intermediate performance, indicating a balance between quality and accessibility, though improvements are needed in both areas.

Readability analysis revealed significant variation across affiliations. While university-affiliated sites were the easiest to understand, commercial sites often required a higher reading level, which could pose challenges for lay readers. These findings mirror prior research indicating that much of the online health information exceeds recommended readability levels for patient education.

Overall, the results indicate that commercial websites generally achieve higher scores on quality measures, such as overall DISCERN ratings and treatment information, but their lower readability may limit accessibility for patients. University/medical centre websites demonstrated the highest readability, making them more suitable for a general audience; however, they scored lower on treatment-related content. Non-profit organisations offered a moderate balance between quality and readability, though improvements are needed in both domains. Clinicians should recognise these differences and guide patients toward resources that are both accurate and easily understood [7].

Limitations and recommendations

This study has several limitations that warrant consideration. Social media platforms, video-based content, and non-English sources were excluded, which may limit the comprehensiveness and generalisability of the findings. Additionally, the results represent a single time point, despite the dynamic nature of online content.

Search engine bias is another potential limitation, as ranking algorithms may prioritise certain websites, thereby influencing the representativeness of the sample. The selection criteria used could also have introduced bias, and although the sample size was relatively substantial, it may not fully reflect the complete spectrum of online information regarding thumb-sucking habits.

The evaluation tools employed, including DISCERN, JAMA benchmarks, and readability indices such as FRES, FKGL, and SMOG, may not fully capture complex aspects of information quality, readability, and user engagement. Furthermore, the absence of specialised content on thumb sucking and the lack of audio formats highlight gaps in available resources, which could affect accessibility and effectiveness.

Future research should address these limitations by broadening the scope to include social media, video-based platforms, and non-English content. Additionally, developing more comprehensive assessment frameworks and exploring the impact of search engine algorithms on information availability and visibility are recommended.

## Conclusions

In conclusion, this study found that the quality and readability of online English-language information on thumb-sucking habits vary across website affiliations. Commercial websites provided higher quality treatment content but were less readable. University/medical centre websites were the most readable but offered lower quality content. Non-profit organisations, representing the majority of sites, balanced quality and readability but lacked sufficient source citation and attribution. These results highlight the need for online health information that is both accurate and accessible. Future studies should include social media and non-English content to provide a more complete picture of available information.
